# The Kidd-Quain Muddle

**Published:** 1881-06

**Authors:** 


APROPOS of the Kid-Quain muddle, we supply our readers with the following sips from *Punch:*

"WHO SHALL DECIDE WHEN DOCTORS," ETC.—The medical profession is *Jenner*-ally represented as disinclined to be hand and glove with Dr. Kidd. This is a *Quain*-t way of putting it.

A RHYME WITHOUT A REASON.—(*By an eminent allopathist*):

I do not like you, Dr. Kidd;
Yet why I don't and never did,
Can't say, but I will bet a "quid"
I do *not* like you, Dr. Kidd.

